# Macrophage-Laden Gold Nanoflowers Embedded with Ultrasmall Iron Oxide Nanoparticles for Enhanced Dual-Mode CT/MR Imaging of Tumors

**DOI:** 10.3390/pharmaceutics13070995

**Published:** 2021-06-30

**Authors:** Yucheng Peng, Xiaomeng Wang, Yue Wang, Yue Gao, Rui Guo, Xiangyang Shi, Xueyan Cao

**Affiliations:** 1 Shanghai Engineering Research Center of Nano-Biomaterials and Regenerative Medicine, College of Chemistry, Chemical Engineering and Biotechnology, Donghua University, Shanghai 201620, China; wang320333@gmail.com (Y.P.); 04200005@sues.edu.cn (X.W.); gaoyue@mail.dhu.edu.cn (Y.G.); ruiguo@dhu.edu.cn (R.G.); 2 Department of Radiology, Shanghai Songjiang District Central Hospital, Shanghai 201600, China; lzyang0816@gmail.com

**Keywords:** gold nanoflowers, ultrasmall iron oxide nanoparticles, multimode imaging, macrophage, tumor diagnosis

## Abstract

The design of multimodal imaging nanoplatforms with improved tumor accumulation represents a major trend in the current development of precision nanomedicine. To this end, we report herein the preparation of macrophage (MA)-laden gold nanoflowers (NFs) embedded with ultrasmall iron oxide nanoparticles (USIO NPs) for enhanced dual-mode computed tomography (CT) and magnetic resonance (MR) imaging of tumors. In this work, generation 5 poly(amidoamine) (G5 PAMAM) dendrimer-stabilized gold (Au) NPs were conjugated with sodium citrate-stabilized USIO NPs to form hybrid seed particles for the subsequent growth of Au nanoflowers (NFs). Afterwards, the remaining terminal amines of dendrimers were acetylated to form the dendrimer-stabilized Fe_3_O_4_/Au NFs (for short, Fe_3_O_4_/Au DSNFs). The acquired Fe_3_O_4_/Au DSNFs possess an average size around 90 nm, display a high r_1_ relaxivity (1.22 mM^−1^ s^−1^), and exhibit good colloidal stability and cytocompatibility. The created hybrid DSNFs can be loaded within MAs without producing any toxicity to the cells. Through the mediation of MAs with a tumor homing and immune evasion property, the Fe_3_O_4_/Au DSNFs can be delivered to tumors more efficiently than those without MAs after intravenous injection, thus significantly improving the MR/CT imaging performance of tumors. The developed MA-mediated delivery system may hold great promise for enhanced tumor delivery of other contrast agents or nanomedicines for precision cancer nanomedicine applications.

## 1. Introduction

Molecular imaging techniques, based on various functional nanoparticles, have attracted increasing attention in the recent development of cancer nanomedicine which is critical for early tumor therapy guidance [1]. Among them, the development of nanoplatforms integrated with different imaging techniques such as computed tomography (CT)[2], ultrasound (US) [3], photoacoustic (PA) [4], and magnetic resonance (MR) [5] imaging represent a competitive strategy for precision cancer diagnoses. Iron oxide NPs have been adopted as negative contrast agents for MR technology due to the T_2_-weighted effect [6]. Au NPs have been broadly used in CT technology and PTT (photothermal therapy) attributing to their intrinsic advantages of an excellent X-ray attenuation feature as well as a near-infrared (NIR) absorption property, respectively [7].

In order to realize the precision molecular imaging, it was desirable to incorporate both Fe_3_O_4_ NPs and Au NPs in one nanoplatform to achieve dual-modal MR/CT imaging [8]. However, to avoid the inaccurate results caused by negative contrast MR images which were hard to be discerned from the tumor site due to dark signal, alternative methods have been explored. Ultrasmall iron oxide (USIO) NPs with a dimension <5 nm have been confirmed and utilized as a promising T_1_-positive contrast agent [9]. Furthermore, the surface modification of USIO with functional moieties could further endow the NPs with targeting or antifouling properties. USIO NPs have also been applied as crosslinkers for alginate nanogels to enhance their r_1_ relaxivity [10]. In addition, different shapes of Au NPs such as Au nanostars (NSs) [11], Au nanorods (NRs) [12], and Au NFs [13] have been confirmed to have good biosafety and high photothermal conversion efficiency [14], employed as a wonderful theranostic reagent for PA technology and PTT. In our previous studies, we have demonstrated that USIO NPs can be embedded within Au NFs to form Fe_3_O_4_/Au NFs with a high r1 relaxivity, leading to the feasibility of multimodal MR/CT/PA imaging and combination PTT/radiotherapy (RT) of tumors [15]. However, considering the fact that NPs are easily non-specifically adsorbed by proteins in the blood vessels, and could be removed rapidly by the reticuloendothelial system (RES) [16], construction of a dual-modal imaging nanoplatform which possesses improved tumor penetration and accumulation abilities is still important and urgent.

To increase the payload at the tumor site, two major strategies are applied, which stand as passive targeting and active targeting. For passive targeting, NPs are directed to the enhanced permeability and retention (EPR) taking advantage of the leaky blood vessels of tumors. However, this targeting ability is mainly affected by the biophysicochemical characteristics of NPs [17], and can be largely weakened due to hypoxia and necrosis of central parts of tumors. While for active targeting, various kinds of targeting ligands are modified to recognize and bind to certain receptors specially expressed on tumor cells [18]. However, the previous study has demonstrated that the peak tumor uptake of the NPs with targeting ligand was <2% of the entire dosage [19]. Moreover, one targeting ligand is often limited to identify several certain types of tumors, thus lacking general applicability [20]. It is also noteworthy that the synthesis of the NPs with targeting ligands usually involve unfavorable complex preparation steps and expensive raw materials [21]. In addition, to overcome RES, a “stealth” strategy of NPs has recently been adopted. This strategy has been proven to have the advantage of avoiding clearance by macrophages; however, at the same time, it owns the disadvantage of suppressing the NPs’ internalization by target cells [22].

Toward these issues, tumor-directed cell-mediated drug delivery systems have become a major focus in cancer theranostics [23]. Specific cells are able to be used as vehicles to convey NPs across the cancer or along the cancer fringe to improve the accumulation of NPs. For instance, red blood cells [24], mesenchymal stem cells (MSCs) [25], neural stem cells [26], and macrophages (MAs) [27] have been regarded as effective tumor-targeting carriers. In addition, these cell-mediated delivery systems play to their inherent characteristics’ strengths, such as having the advantages of non-tumorigenic, low immunogenicity, and being able to cross certain physiological barriers in the body [28]. The FDA has approved a stem cells carrier to be used in clinical trials for glioma treatment. Among these cell vehicles [29], macrophages possess the unique advantages of minimized phagocytosis and extraction from peripheral blood, which is particularly critical in drug delivery. Previously, we have confirmed that MSCs-mediated delivery systems of nanogels with Fe_3_O_4_ NPs improved the magnetic MR effect in breast and glioma tumor models in comparison with free nanogels loaded with Fe_3_O_4_ NPs [30]. Recently, Guo et al. [31] treated ovarian cancer mice with macrophage-loaded doxorubicine (DOX) and found that macrophages entered the tumor tissue and effectively delivered the drug directly to cancer cells through a tunneling nanotube pathway. Moreover, it was found that the chemokine receptors CCR2 and CCR4 of drug-loaded macrophages were highly expressed, which enhanced the tropism of macrophages. Hence, it is logical to assume that USIO NPs could be loaded onto Au NFs and carried by cancer tropic macrophagocytes to reach tumors for better cancer MR/CT image formation.

**Scheme 1:**
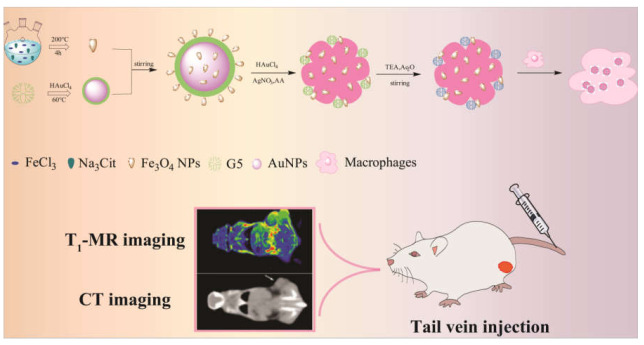
Schematic illustration of the preparation of MA@Fe_3_O_4_/Au DSNFs for dual-modal imaging of tumors.

In the present study, our team developed a novel MA-laden nanomedicine platform to realize improved cancer MR/CT imaging. First, citrate-stabilized USIO NPs and Amine-terminated G5 PAMAM dendrimers-stabilized gold nanoparticles (Au DSNPs) were prepared by a solvothermal route mentioned and self-reduction. Then, Au DSNPs and USIO NPs constituted the seed particles via a 1-(3-dimethylaminopropyl)-3-ethylcarbodiimide hydrochloride (EDC)-mediated covalent reactive process and were used to generate Au NFs. After that, we acetylated the amino group at the terminal of PAMAM to change the surface potential of the Fe_3_O_4_/Au DSNFs. The generated Fe_3_O_4_/Au DSNFs were loaded in MA for further conveyance to cancers posterior to intravenous injection for MR/CT imaging of tumors (Scheme 1). The generated Fe_3_O_4_/Au DSNFs were completely characterized by numerous approaches. The cytocompatibility and uptake efficiency via MA were comprehensively assessed. The underlying value of the MA loaded with the Fe_3_O_4_/Au DSNFs for improved cancer MR/CT imaging was subsequently examined in vivo by mice breast cancer models. For all we know, this is the first research on the development of the MA-laden Fe_3_O_4_/Au DSNFs platform for enhanced tumor MR/CT image formation.

## 2. Materials and Methods

### 2.1. Synthesis of Fe_3_O_4_/Au DSNFs

Firstly, the ultrasmall iron oxide was acquired by a solvothermal route introduced in our past research [30]. Meanwhile, G5 PAMAM Au DSNPs were prepared by the self-reduction method [11]. We obtained Fe_3_O_4_/Au DSNFs through an EDC-mediated covalent reaction with Au DSNPs and EDC-activated USIO NPs based on literature [15]. Briefly, the carboxyl groups of USIO NPs were activated with EDC, then the activated USIO NPs were added dropwise into the solution of Au DSNPs with amino groups on the surface, and finally the Fe_3_O_4_/Au DSNPs was generated through covalent bonding. The terminal amines of the PAMAM were finally treated with acetyl to reduce the positive surface charge of the hybrid nanomaterials. The generated Fe_3_O_4_/Au DSNFs have been characterized by different techniques, such as transmission electron microscopy (TEM), dynamic light scatter (DLS), and UV-vis spectra.

### 2.2. In Vitro Cytotoxicity and Cellular Uptake Assays

Mouse monocyte macrophages (Raw264.7) were incessantly cultivated in the DMEM intermediate. Cytotoxicity of the Fe_3_O_4_/Au DSNFs relative to Raw264.7 cells was detected using a CCK-8 assay according to the literature [27]. The transwell experiment was carried out to verify whether the tumor-tendency of macrophages changed after incubation with Fe_3_O_4_/Au DSNFs for 18 h. The cell uptake of the Fe_3_O_4_/Au DSNFs was evaluated as well by the ICP-OES method and Prussian blue dyeing. The phenotype of Raw264.7 cells after incubating with Fe_3_O_4_/Au DSNFs for 6 h were evaluated by flow cytometry (BD FACS Calibur, Franklin Lakes, NJ, USA).

### 2.3. In Vivo MR and CT Imaging of Breast Tumor Model

The mouse breast tumor model was built in male 4~6 weeks old ICR mice for MR and CT imaging. All mice were injected with 2 × 10^6^ 4T1 cells into the right legs to form the breast cancer models. Until the cancer dimensions of mice registered 0.45~0.75 cm^3^, macrophagocytes mediated Fe_3_O_4_/Au DSNFs ([Fe] = 100 μg, in 0.2 mL PBS) were injected into all mice and similar dosages of free Fe_3_O_4_/Au DSNFs were injected into the control group for MR image formation. In addition, macrophages mediated Fe_3_O_4_/Au DSNFs ([Au] = 3 mM, in 0.2 mL PBS) were injected into all mice and a similar dosage of free Fe_3_O_4_/Au DSNFs were injected into the control group for CT imaging. The in vivo breast cancer imaging studies were measured by the CT and MR method. Afterwards, the CT and T_1_-weighted MR images of all mice were acquired prior to and posterior to the injection of the MA@Fe_3_O_4_/Au DSNFs or Fe_3_O_4_/Au DSNFs at diverse time points.

## 3. Results and Discussion

### 3.1. Characterization of Fe_3_O_4_/Au DSNFs

**Figure 1:**
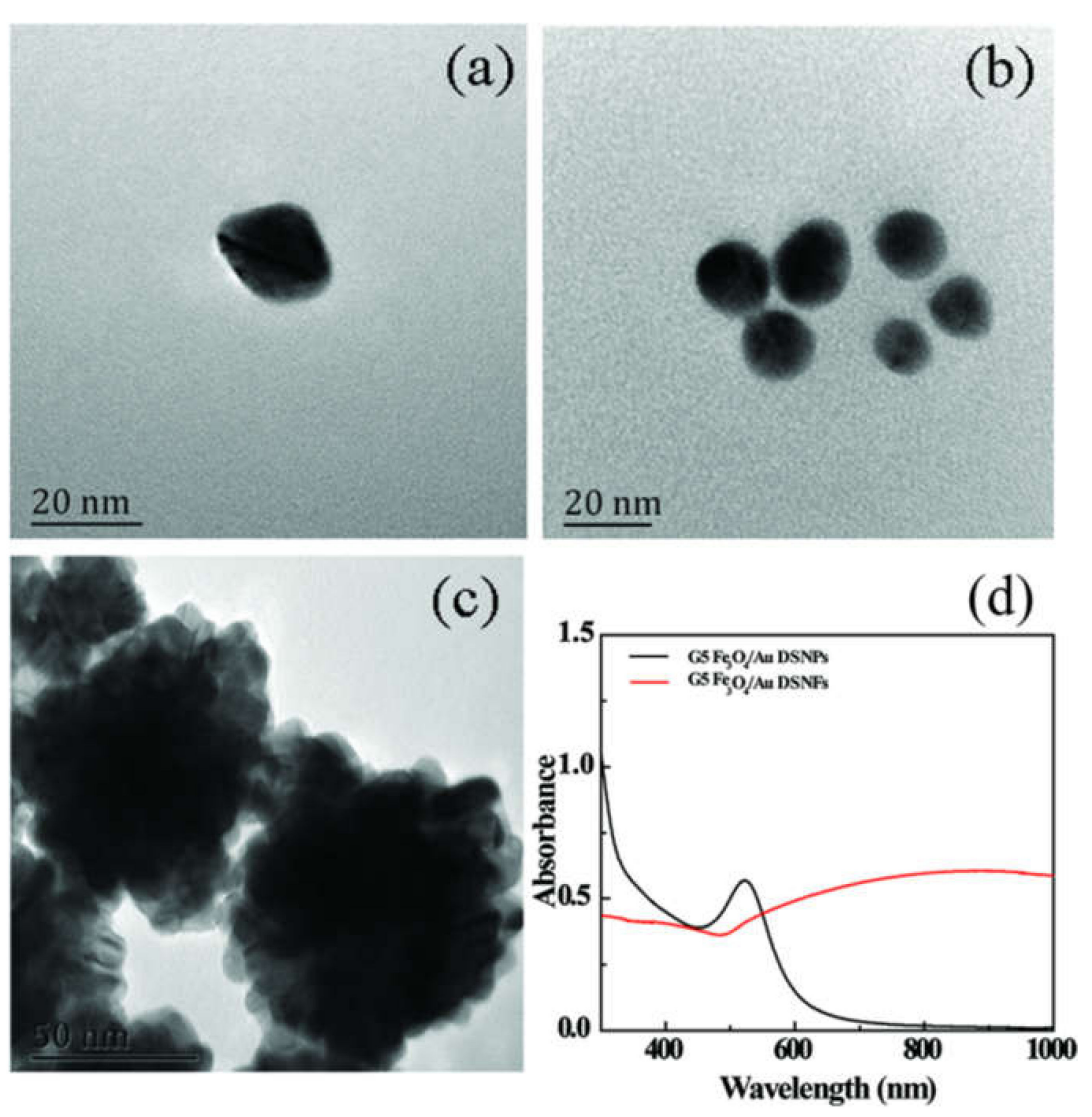
Structural characterization of Fe_3_O_4_/Au DSNFs. (**a**) TEM images of Au NPs, (**b**) TEM images of Fe_3_O_4_/Au DSNPs, (**c**) TEM images of Fe_3_O_4_/Au DSNFs, (**d**) UV-vis spectrum of Fe_3_O_4_/Au DSNPs and Fe_3_O_4_/Au DSNFs.

USIO NPs were first synthesized to be used as seed particles. The results of TEM showed that the diameter of formed USIO NPs is 2.6 ± 0.61 nm (Figure S1a,b). Amine-terminated G5 PAMAM dendrimers-stabilized gold nanoflowers were prepared by a self-reduction method, which displayed a size around 20 nm (Figure 1a). Meanwhile, the particles of Fe_3_O_4_/Au DSNPs at the size of 15 nm (Figure 1b) were obtained by EDC-activated USIO NPs which reacted with Au DSNPs by an amide linkage. The size of the Fe_3_O_4_/Au DSNPs measured by TEM is smaller than the individual Au DSNPs, indicating the remarkable interaction between Au DSNPs and USIO NPs, in accordance with the results reported by previous literature [15]. After Fe_3_O_4_/Au DSNPs merge with an Au growth solution, their sizes gradually grow to 98 nm. It is clear to see the nanoflower structure as shown in Figure 1c.

The zeta potential and hydrodynamic size of USIO NPs, Au DSNPs, Fe_3_O_4_/Au DSNPs, and Fe_3_O_4_/Au DSNFs were subsequently tested by DLS. Obviously, the zeta potential of the USIO NPs and Au DSNPs is −24.6 ± 3.1 mV and 23.8 ± 3.5 mV, respectively. The change is supposed to be due to the rich citric acid carboxyl and PAMAM terminal amines on their surface. The surface potentials of the Fe_3_O_4_/Au DSNPs were measured to be 19.2 ± 2.2 mV, because the hybrid reaction neutralized partial terminal amines of Au DSNPs. In order to shield the residual primary amines of G5 dendrimers, the formed Fe_3_O_4_/Au DSNPs were acetylated, leading to a reduction of zeta potential to 11.2 ± 2.7 mV. The hydrodynamic size of USIO NPs, Au DSNPs, seed particles, and Fe_3_O_4_/Au DSNFs is 27.5 ± 2.4 nm, 30.7 ± 7.2 nm, 28.2 ± 3.1 nm, and 265.3 ± 6.9 nm, respectively. Due to the aggregation nature of the nanoparticles in water, an increasing hydrodynamic size was observed in aqueous solutions compared to the data obtained from TEM. However, the hydrodynamic sizes shared a similar variation trend with the TEM results, indicating that the NFs have been successfully prepared.

The structure of Fe_3_O_4_/Au DSNFs was confirmed by UV-vis (Figure 1d). The results showed that the characteristic ultraviolet absorptions of G5 PAMAM gold particles and Fe_3_O_4_/Au DSNFs fall at about 520 nm and 820 nm, respectively. It is proven that the Fe_3_O_4_/Au DSNFs were successfully constructed, which was consistent with the previous literature [15]. In order to assess the steadiness of the Fe_3_O_4_/Au DSNFs, we surveyed the size and polymer dispersity index (PDI) of Fe_3_O_4_/Au DSNFs at diverse concentrations within 14 days. As displayed in Figure S2, no apparent changes could be found, suggesting its good colloidal stability and dispersity.

### 3.2. In Vitro T_1_-Weighted MR Images

**Figure 2:**
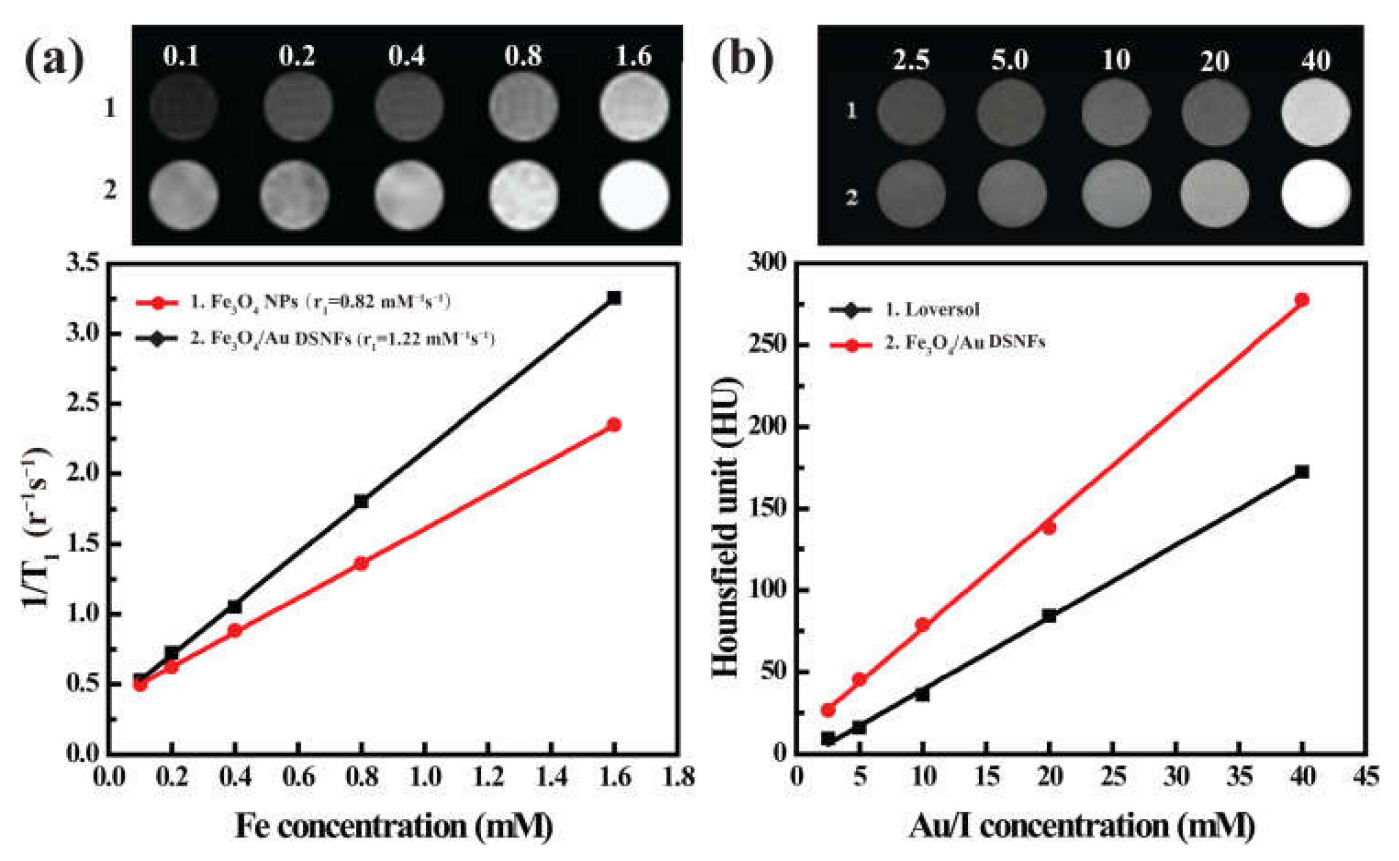
CT and MR imaging properties of the Fe_3_O_4_/Au DSNFs. (**a**) T_1_-weighted MR images and linear fitting of 1/T_1_ of the Fe_3_O_4_ NPs and Fe_3_O_4_/Au DSNFs at diverse Fe concentrations (1 and 2 refers to USIO NPs and NFs, separately). TE = 12 ms, TR = 377 ms, and ST = 2.0. (**b**) CT images and CT values (HU) of Loversol and Fe_3_O_4_/Au DSNFs with diverse concentrations of the radiodense element of Au or I (1 and 2 refers to Omnipaque and NFs, respectively).

As it contains both the radiodense element (Au) and T_1_ positive agents (Fe_3_O_4_), Fe_3_O_4_/Au DSNFs can be utilized for CT and MR imaging. To verify the MR imaging potential of the USIO NPs and Fe_3_O_4_/Au DSNFs in vitro, their r_1_ relaxivity were tested. As shown in Figure 2a, the r_1_ relaxivity of the USIO NPs and Fe_3_O_4_/Au DSNFs were computed to be 0.82 mM^−1^ s^−1^ and 1.22 mM^−1^ s^−1^, separately. It is distinctly shown that the r_1_ relaxivity of Fe_3_O_4_/Au DSNFs is 1.47 times greater than that of the USIO NPs. It is attributed to the fact that USIO NPs were sufficiently dispersed by G5 dendrimer-stabilized Au NPs, while the NFs structure by the seed-mediated approach does not appear to cause remarkable clustering of USIO NPs. Meanwhile, it is clear that USIO NPs and Fe_3_O_4_/Au DSNFs have shown excellent MR imaging ability, which is strengthened with the rise of Fe concentration. The CT imaging property of Fe_3_O_4_/Au DSNFs was detected, and a clinical CT contrast agent Loversol was tested as the positive control (Figure 2b). CT images verify that the HU of Loversol and Fe_3_O_4_/Au DSNFs increase significantly with the increase of concentration Au and I. Moreover, at the same concentration of Au and I, Fe_3_O_4_/Au DSNFs have a higher attenuation coefficient and a more significant imaging effect. The results demonstrated that the synthesized Fe_3_O_4_/Au DSNFs can be used as a potential multimodal contrast agent in MR and CT molecular imaging diagnoses.

### 3.3. In Vitro Cytotoxicity and Cellular Uptake Assays

**Figure 3:**
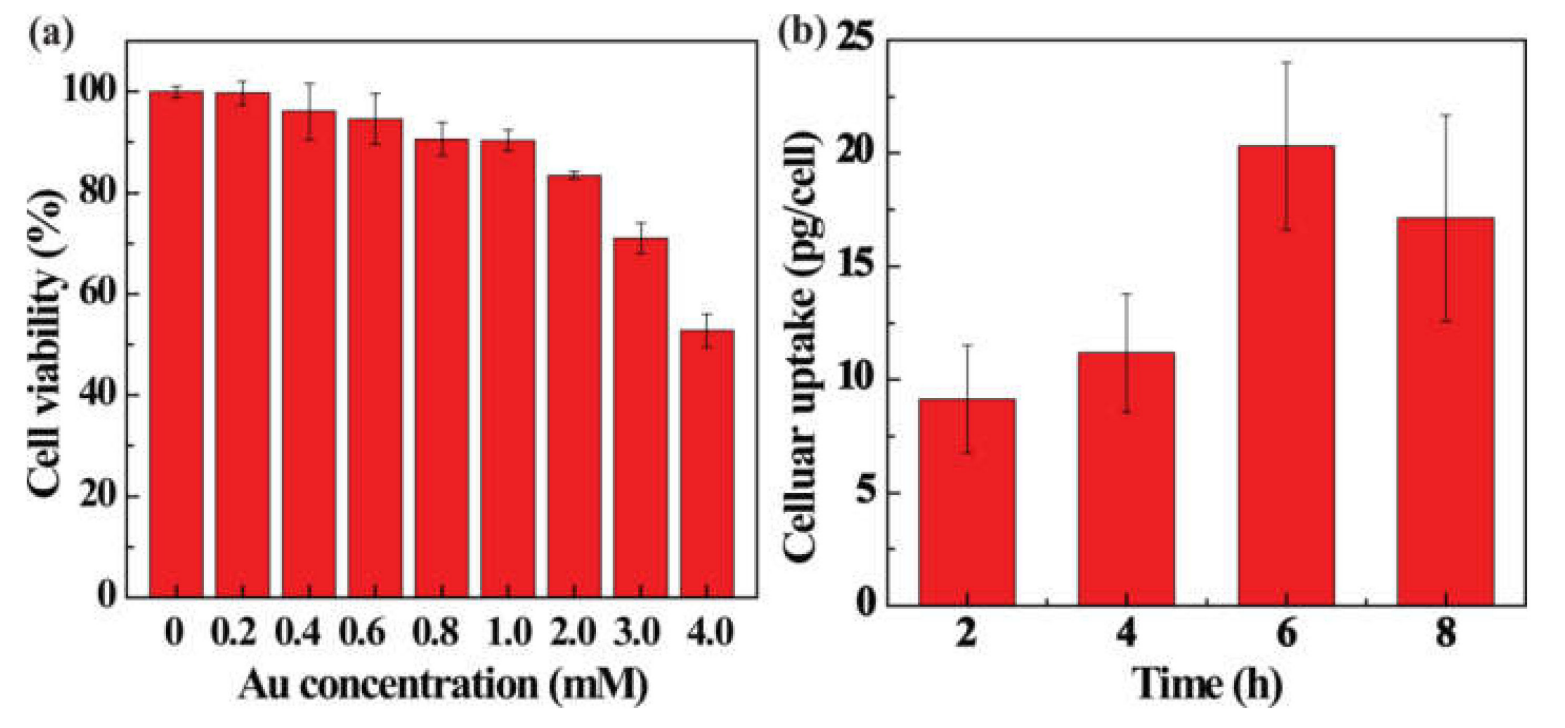
(**a**) Cell viability of Fe_3_O_4_/Au DSNFs cultured with Raw264.7 cells in different Fe concentration. (**b**) Cellular uptake of Fe_3_O_4_/Au DSNFs with MAs for 2, 4, 6, and 8 h.

The influence of materials on cell vitality is vital for subsequent in vivo imaging applications. Therefore, the cytotoxicity of Fe_3_O_4_/Au DSNFs was first explored before the in vivo multimode imaging application. The viability of Raw264.7 cells was implemented by CCK-8 assay after a treatment with the Fe_3_O_4_/Au DSNFs for 24 h (Figure 3a). It is obvious that the cell viability gradually decreased with the rise of Au quantity. However, the viability was still higher than 72% when the Au concentration reached 3 mM, implying that the Fe_3_O_4_/Au DSNFs register an acceptable cytocompatibility when the Au concentration ranges from 0 mM to 3 mM. To find the cellular uptake efficiency of Fe_3_O_4_/Au DSNFs by Raw264.7 cells, the Au uptake of Raw264.7 cells co-cultured with the Fe_3_O_4_/Au DSNFs ([Au] = 3 mM) from 2 h to 8 h was assessed in terms of quantity inductively coupled plasma-optical emission spectroscopy (ICP-OES) (Figure 3b). The results show the phagocytic capacity is the highest when Raw264.7 cells were cultivated with Fe_3_O_4_/Au DSNFs for 6 h. Furthermore, with the increase of Fe concentration, the darker dyeing could be found after the cells were treated with the Fe_3_O_4_/Au DSNFs based on Fe uptake qualitatively surveyed by Prussian blue staining (Figure S3). The results confirmed that the Fe_3_O_4_/Au DSNFs can be easily taken up by Raw264.7 cells, which was useful to enhance the multimode imaging ability of the Fe_3_O_4_/Au DSNFs.

**Figure 4:**
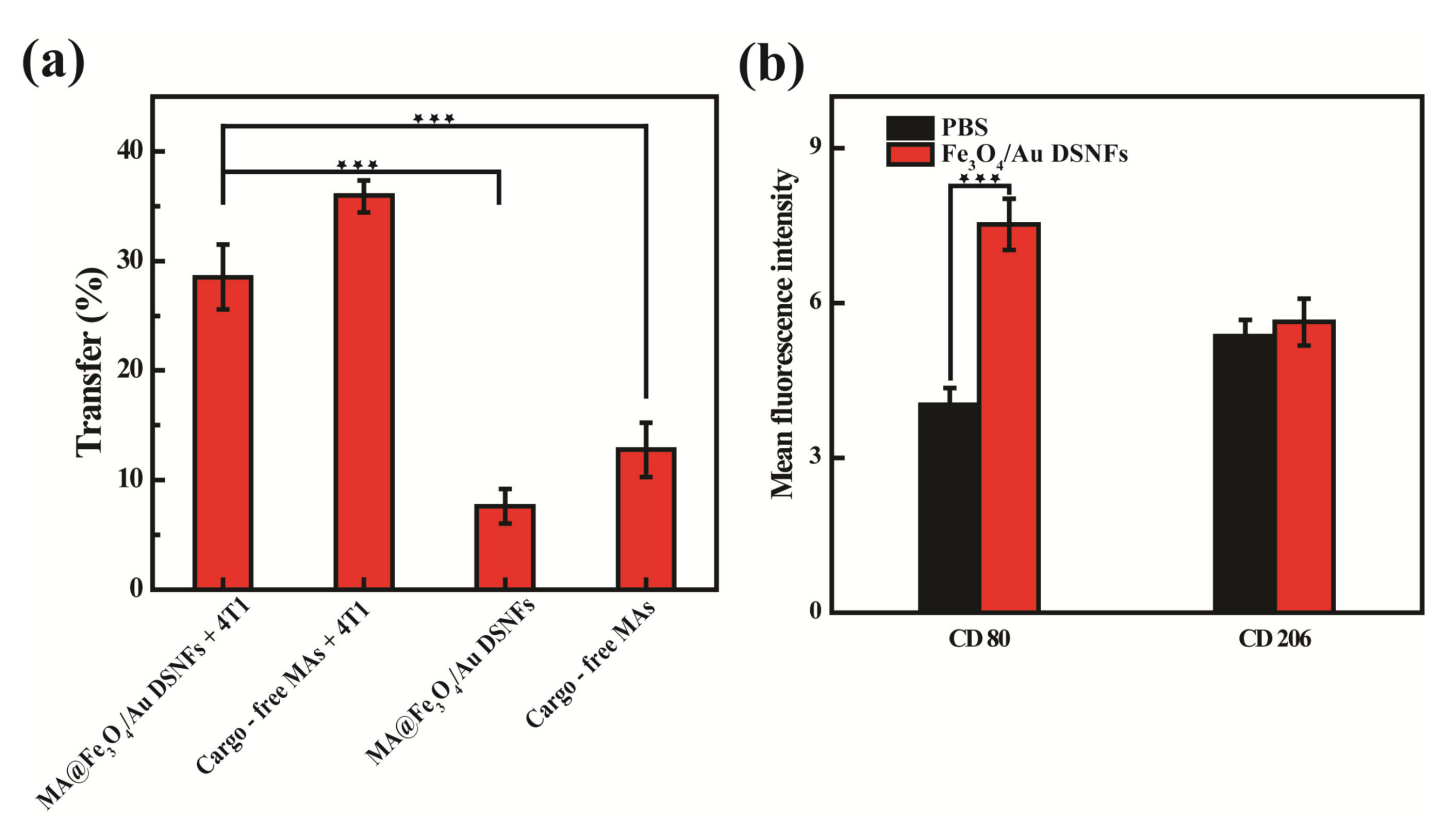
(**a**) Cell migration rates of Raw264.7 cells to 4T1 cells treated with MA@Fe_3_O_4_/Au DSNFs with 4T1 cell-guided, MA with 4T1 cell-guided, MA@Fe_3_O_4_/Au DSNFs without 4T1 cell-guided, and MA without 4T1 cell-guided for 18 h. (**b**) Quantification of CD80 and CD206 expression via flow cytometry for Raw264.7 cells incubating with and without Fe_3_O_4_/Au DSNFs in the in vitro experiment.

Tumor tropism is a typical peculiarity of macrophages because it can be guided by chemokines and cytokines to the inflammation site. The transwell migration experiment was designed to verify whether the functional properties of macrophages have changed after Raw264.7 cells were incubated with the Fe_3_O_4_/Au DSNFs for 18 h (Figure 4a). In this experiment, 4T1 cells were utilized. It can be seen that the presence of 4T1 cells has a significant effect on the migration rate of Raw264.7 cells, which is about three times than that in the absence of 4T1 cells. Although the cell migration rate was slightly reduced after co-incubation with Fe_3_O_4_/Au DSNFs compared with free MAs, the tumor tropism of MA@Fe_3_O_4_/Au DSNFs was still significant. It indicates that the activities and roles of Raw264.7 cells are not influenced posterior to the uptake of the Fe_3_O_4_/Au DSNFs.

In addition, we must consider that macrophages are easy to differentiate into various phenotypes that exacerbate or resolve the disease. Previous studies have confirmed that iron can activate the differentiation of macrophages to the M1 phenotype [32]. Therefore, it is necessary to detect the activation states and the biomarkers of macrophage phenotypes after the incubation of Fe_3_O_4_/Au DSNFs. As we know, M1 macrophages could be identified by certain special surface antigens such as CD80 and CD86, while CD206 antigens are expressed in M2 macrophages. Thus, Raw264.7 cells incubated with Fe_3_O_4_/Au DSNFs for 6 h were analyzed by specific M2 and M1 macrophages antigens CD206 (Figure S4a) and CD80 (Figure S4b) by flow cytometry. For quantitative results, the expression of CD206 by Raw264.7 cells with and without Fe_3_O_4_/Au DSNFs are 5.36 ± 0.31 and 5.63 ± 0.45, respectively (Figure 4b). The similar results indicated that the loading of Fe_3_O_4_/Au DSNFs did not stimulate macrophages to the M2 phenotype. After incubation with and without Fe_3_O_4_/Au DSNFs the expression level of CD80 are 7.52 ± 0.5 and 4.03 ± 0.32, respectively (Figure 4b), proving that Fe_3_O_4_/Au DSNFs activate macrophages to the M1 phenotype. It suggests that the performance of macrophages differentiate toward the M1 phenotype after uptake of the Fe_3_O_4_/Au DSNFs, possibly exhibiting tumoricidal activity. Thus, the above results also show that the MA@Fe_3_O_4_/Au DSNFs have potential applications in cancer therapy.

### 3.4. In Vivo MR and CT Imaging of Breast Tumor Model

**Figure 5:**
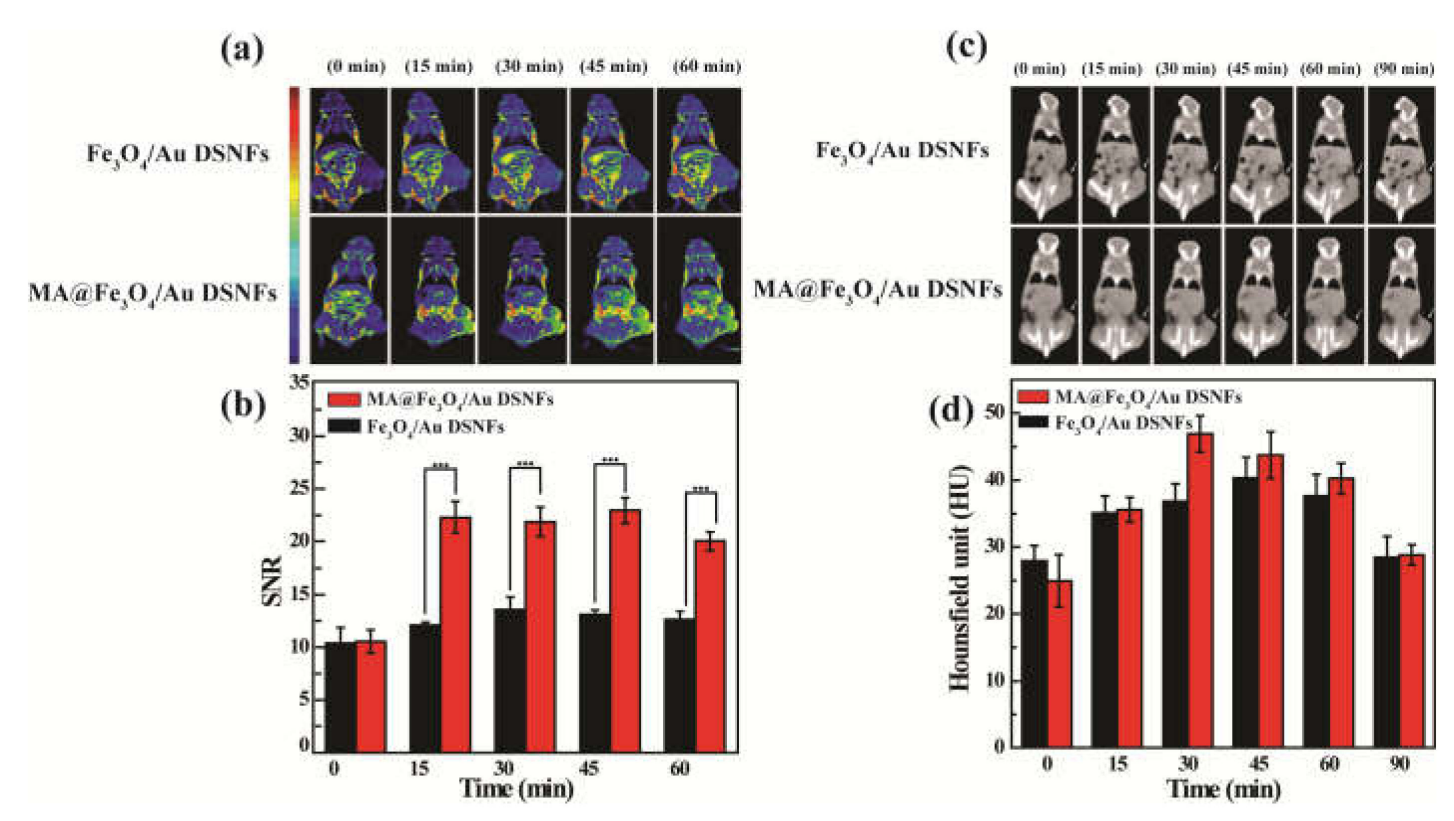
In vivo tumor MR and CT imaging. (**a**) In vivo T_1_-weighted MR images and (**b**) the corresponding MR SNR of the 4T1 tumor anterior to injection and at diverse time points post-injection of Fe_3_O_4_/Au DSNFs and MA@Fe_3_O_4_/Au DSNFs ([Fe] = 100 μg, in 0.2 mL PBS for every mouse), separately (mean ± S.D., *n* = 3). The MR signal intensity of blank (air) was utilized as the background (noise). TE = 12 ms, TR = 377 ms, and ST = 2.0. (**c**) CT images and (**d**) corresponding CT values of the 4T1 cancer in ICR mice before and at diverse time points post intravenous injection of the Fe_3_O_4_/Au DSNFs and MA@Fe_3_O_4_/Au DSNFs ([Au] = 0.04 M, in 200 µL PBS for every mouse).

Next, we investigated the capability of Fe_3_O_4_/Au DSNFs for in vivo duo-modal MR/CT imaging of a subcutaneous tumor model (Figure 5). Free Fe_3_O_4_/Au DSNFs without Raw264.7 cells mediated were taken as the control group. It can be seen that the tumor regions are progressively illuminated over time posterior to injection of Fe_3_O_4_/Au DSNFs and MA@Fe_3_O_4_/Au DSNFs (Figure 5a). Moreover, we obtained the best MR imaging performance at 30 min after injection, which may be attributed to the largest cumulation of MA@Fe_3_O_4_/Au DSNFs in the tumor region. For contrast, merely a little rise of the MR signal intensity could be detected in Fe_3_O_4_/Au DSNFs samples. Most importantly, even during metabolism, we noted that MA@Fe_3_O_4_/Au DSNF could still provide premium imaging of the tumor area after 60 min posterior to injection. It is supposed that macrophages can target to the tumor region, resulting in the better accumulation of MA@Fe_3_O_4_/Au DSNFs than free Fe_3_O_4_/Au DSNFs. Besides, the released Fe_3_O_4_/Au DSNFs via exocytosis could be incessantly utilized for MR imaging of tumors until they are metabolized out of the body, thereby displaying a greater retention time than free Fe_3_O_4_/Au DSNFs. The quantity outcomes of MR SNR data were evaluated at diverse time points as well (Figure 5b). Within 15–45 min posterior to tail vein injection, the cancer SNR value of MA@Fe_3_O_4_/Au DSNFs is around 1.5–1.9 times greater than that treated with Fe_3_O_4_/Au DSNFs (*p* < 0.001). Those results display that the MA@Fe_3_O_4_/Au DSNFs demonstrate a synergistically improved T_1_ imaging effect in contrast to Fe_3_O_4_/Au DSNFs.

Likewise, due to the outstanding effect of Au on CT imaging, we subsequently studied the performance of MA@Fe_3_O_4_/Au DSNFs for in vivo CT imaging (Figure 5c). As we could see, macrophages facilitated targeted delivery of Fe_3_O_4_/Au DSNFs to the tumor region, thereby improving the effect of CT imaging compared with free Fe_3_O_4_/Au DSNFs. The highest resolution CT imaging of a tumor area of MA@Fe_3_O_4_/Au DSNFs was obtained after 30 min from tail vein injection. Quantitatively, the CT value of tumor site reached the summit value at 30 min posterior to intravenous injection of MA@Fe_3_O_4_/Au DSNFs which registered lesser time consumption in comparison with free Fe_3_O_4_/Au DSNFs, and the summit value (46 HU) is 1.2 times greater than that of free Fe_3_O_4_/Au DSNFs (39 HU). The entire outcomes validate that a macrophages-laden Fe_3_O_4_/Au DSNFs delivery nano-system could enhance the accumulation and prolong the retention time of the Fe_3_O_4_/Au DSNFs in a tumor region. Accordingly, it could be applied for enhanced MR/CT imaging of tumors.

## 4. Conclusions

In summary, we developed a novel targeted diagnostic platform based on the MA-laden delivery of Fe_3_O_4_/Au DSNFs for enhanced multimodal imaging of tumors. The prepared Fe_3_O_4_/Au DSNFs register a size of 98 nm resembling our past research, and are proven to be uniform, colloidally stable, and cytocompatible. It displayed predominant performance on CT and MR imaging with relatively high r_1_ relaxivity (1.22 mM**^−^**^1^ s**^−^**^1^), and could be well taken up by macrophages with little influence on cell viability so that MA@Fe_3_O_4_/Au DSNFs could be successfully utilized in the in vivo CT and MR molecular imaging of the breast cancer model. The entire outcome concluded that MA-mediated Fe_3_O_4_/Au DSNFs are possible to be used as an unusual multimodal contrast agent for tumor diagnosis. Further studies on the PA performance and photothermal treatment effect of the developed MA-laden Fe3O4/Au DSNFs should be conducted to build multifunctional nanoplatforms for precise tumor treatment.

## Data Availability

Not applicable.

## Supplementary Materials

The following are available online at https://www.mdpi.com/article/10.3390/pharmaceutics13070995/s1, Figure S1: (a) TEM image and (b) the mean dimension distribution column diagram of USIO NPs; Figure S2: Digital photos of Fe_3_O_4_/Au DSNFs with diverse molecular rates of Fe/Au (from 1:1 to 6:1), (a) 3 d (b) 5 d (c) 7 d; Figure S3: MA@Fe_3_O_4_/Au DSNFs with different Fe concentrations (0, 0.44, 0.88, and 1.76 mM) to incubate with Raw264.7 cells for 6 h and stain with Prussian blue reagent; Figure S4: Identification of special CD markers in Raw264.7 cells via flow cytometric method. Raw264.7 cells were dyed with fluorescence conjugated antibodies fluorescein phycoerythrin conjugated rat anti mouse (a) CD206 and (b) CD80.
