# Supplementary material for: Macrophage-Laden Gold Nanoflowers Embedded with Ultrasmall Iron Oxide Nanoparticles for Enhanced Dual-Mode CT/MR Imaging of Tumors
Source: Pharmaceutics. 2021 Jun 30;13(7):995. doi: 10.3390/pharmaceutics13070995 (PMC8308993; doi:10.3390/pharmaceutics13070995)
Supplement: Supplementary file 1 [file pharmaceutics-13-00995-s001.zip › pharmaceutics-1223541-supplementary.pdf]

pharmaceutics

Supplementary Materials: Macrophage-Laden Gold Nanoflowers Embedded with Ultrasmall Iron Oxide Nanoparticles for Enhanced Dual-Mode CT/MR Imaging of Tumors

Yucheng Peng, Xiaomeng Wang, Yue Wang, Yue Gao, Rui Guo, Xiangyang Shi and Xueyan Cao

Materials

Ethylenediamine core G5.NH2 PAMAM dendrimers were purchased from Dendritech (Midland, MI, USA). 1-(3-Dimethylaminopropyl)-3-ethylcarbodiimide hydrochloride (EDC.HCl) and N-hydroxysuccinimide (NHS) were from GL Biochem. (Shanghai, China). Iron(III) chlorideanhydrous, $HAuCl_4 \cdot 4H_2O$, silver nitrate ($AgNO_3$), ascorbic acid (AA), dimethyl sulfoxide (DMSO), diethylene glycol (DEG), trisodium citrate dihydrate ($Na_3Cit \cdot 2H_2O$), sodium acetate anhydrous (NaOAc), and other agents were from Sinopharm Chemical Reagent Ltd. (Shanghai, China). Raw264.7 cells (a murine macrophage cell line) and 4T1 cells (a murine breast cancer cell line) were from Institute of Biochemistry and Cell Biology (the Chinese Academy of Sciences, Shanghai, China). Dulbecco's modified Eagle's medium (DMEM), fetal bovine serum (FBS), trypsin, penicillin, and streptomycin were obtained from Hangzhou Jinuo Biomedical Technology (Hangzhou, China). The cell-counting kit-8 (CCK-8) was provided by 7Sea Biotech. Co., Ltd. (Shanghai, China). Water used in all experiments was purified using a Milli-Q Plus 185 water purification system (Millipore, Bedford, MA, USA) with a resistivity higher than 18 MΩ·cm. Regenerated cellulose dialysis membranes with a molecular weight cut-off (MWCO) of 8000–14,000 were acquired from Beijing Secoma Biotechnology Corporation (Beijing, China).

Synthesis of USIO NPs

USIO NPs were synthesized according to our previous work [1]. In brief, $FeCl_3$ (4 mmol) was dissolved into 40 mL of diethylene glycol (DEG) under stirring and then $Na_3Cit \cdot 2H_2O$ (1.6 mmol) was added into the above solution at 80 °C and reacted for 2 h, followed by the addition of NaOAc (12 mmol) to form a clear solution. After that, the mixture was transferred to a stainless-steel reaction kettle with a volume of 50 mL and reacted at 200 °C for 4 h. Afterwards, the black product was cooled down to an ambient temperature, collected by centrifugation (8500 rpm, 15 min), and purified with anhydrous ethanol 4 times. The resulting precipitate was dried at 60 °C and stored at −20 °C before use.

Synthesis of Au DSNPs

Au DSNPs were prepared according to the literature [2]. In brief, G5 dendrimers (0.002 mmol) were dissolved in water (30 mL) and the solution was preheated in a water bath at 60 °C for 30 min. Then, $HAuCl_4 \cdot 4H_2O$ (0.073 M, in 353 μL water) was added into the above solution under magnetic stirring at 60 °C for 3 h. The color of the reaction mixture gradually changed from auratus to lilac. Afterwards, the solution was cooled down to room temperature and lyophilized to get the product of Au DSNPs.

Synthesis of Fe_3O_4/Au DSNPs

The surface carboxyl groups of USIO NPs (56 mg, in 5 mL DMSO) were activated by EDC (144 mg, in 2 mL DMSO) and NHS (70 mg, 2 mL DMSO) for 3 h under stirring. After

activation, the product was dialyzed against water (6 times, 2 μL) using a dialysis membrane with an MWCO of 3000 for 3 days, followed by lyophilization to obtain the product of carboxyl-activated ultrasmall Fe_3O_4 NPs.

The activated USIO NPs (8.88 mg) were added into the Au DSNP solution (28 mg, 10 mL in water) and the reaction was continued for 3 days under magnetic stirring to acquire the Fe_3O_4/Au DSNPs complexes. After that, the product was dialyzed against water (6 times, 2 μL) using a dialysis membrane with an MWCO of 3000 for 3 days to purify the Fe_3O_4/Au DSNPs complexes. The final Fe_3O_4/Au DSNPs complexes were dispersed into 10 mL of water before use.

Synthesis of Fe_3O_4/Au DSNFs

The above Fe_3O_4/Au DSNPs complexes (100 mL) added into an aqueous solution of $HAuCl_4$ (0.25 mM, 10 mL) under stirring, then $AgNO_3$ (2 mM, 100 μL in water) and AA (0.1 M, 50 μL in water) were immediately added into the above solution. The solution was stirred for 2 h to form Au DSNFs embedded with USIO NPs. After that, the remaining dendrimer terminal amine groups on the surface of the Au DSNFs were completely acetylated according to the literature [3]. Briefly, 3.8 μL of triethylamine was added to the aqueous solution of Fe_3O_4/Au DSNFs, and the solution was adequately mixed for 30 min. Then, 2.1 μL of acetic anhydride was added into the above solution under vigorous magnetic stirring for 24 h. Finally, the above mixture was centrifuged at 8500 rpm for 20 min and redispersed into water at least three times and the purified particles were lyophilized to obtain the final Fe_3O_4/Au DSNFs. For comparison, Au DSNFs without USIO NPs were also prepared under the same conditions.

Characterization Techniques

Transmission electron microscopy (TEM) was carried out by JEOL 2010F electron microscope (Tokyo, Japan) at an operating voltage of 200 kV. A typical sample with a volume of 6 μL in water was deposited onto a carbon-coated copper grid and air dried before observation. Dynamic light scattering (DLS) and zeta potential measurements were performed using a Malvern Zetasizer (Nano ZS model ZEN3600, Worcestershire, UK) equipped with a standard 633 nm laser. Samples were dispersed in water with a concentration of 1 mg/mL before measurements. A thermal gravimetric analysis (TGA) was executed using a TG 209 F1 thermal gravimetric analyzer (NETZSCH Instruments Co., Ltd., Selb/Bavaria, Germany) at a heating rate of 20 °C/min and in a temperature range of 30-700°C under N_2 atmosphere. UV-vis spectra were collected using a Lambda 25 UV-vis spectrophotometer (Perkin Elmer, Boston, MA, USA). Each sample was dispersed in water and placed into a cuvette (1 mL) before measurements. The Fe and Au composition of the formed USIO NPs, Au DSNPs, Fe_3O_4/Au DSNPs complexes, or Fe_3O_4/Au DSNFs was determined by Leeman Prodigy inductively coupled plasma-optical emission spectroscopy (ICP-OES, Hudson, NH, USA).

MR/CT Imaging Performance of the Fe_3O_4/Au DSNFs

T_1 magnetic resonance (MR) relaxometry was performed by a 0.5 T NMI20 Analyzing and Imaging System (Shanghai NIUMAG Corporation, Shanghai, China). The parameters were set as follows: TR = 400 ms, TE = 20 ms, resolution = 156 mm × 156 mm, and section thickness = 0.5 mm. The r_1 relaxivity was obtained through linear fitting of the inverse T_1 relaxation time ($1/T_1$) as a function of Fe concentration. Both USIO NPs and Fe_3O_4/Au DSNFs with the same Fe concentrations (0.1–1.6 mM) were determined. Concentration-dependent brightening effects were observed by T_1-weighted MR images.

The GE LightSpeed VCT Imaging System (GE Medical Systems, Milwaukee, WI, USA) was applied for CT scanning at 100 kV, 80 mA, and a slice thickness of 0.625 mm. Contrast enhancement was determined in Hounsfield units (HU) for each sample with different Au concentrations. The CT imaging performance of Fe_3O_4/Au DSNFs was compared to a clinical contrast agent (Loversol) at different molar concentrations of Au or iodine (2.5–40 mM).

In vitro Cytotoxicity and Cellular Uptake Assays

Raw264.7 cells were continuously cultured and passaged in a regular DMEM medium added with 10% heat-inactivated FBS, 1% penicillin and streptomycin in a 37 °C incubator with 5% CO_2. Cytotoxicity of the Fe_3O_4/Au DSNFs relative to Raw264.7 cells was detected using the CCK-8 assay according to our previous study [4]. The cellular uptake of the Fe_3O_4/Au DSNFs was also assessed by a Leeman Prodigy ICP-OES System (Hudson, NH, USA) and Prussian blue staining.

Macrophage Cellular Uptake Assays

The uptake of the Fe_3O_4/Au DSNFs by MA was investigated by an ICP-OES analysis. Raw 264.7 cells were seeded in 12-well plates at a density of 2×10^5 cells per well in 1 mL DMEM medium and incubated at 37 °C and 5% CO_2. After incubation, the medium was replaced with 1 mL fresh medium containing PBS (control), and Fe_3O_4/Au DSNFs at different Fe concentrations (0~0.2mM, respectively). The cells were incubated for another 4~8 h, after counting the cell number in the cell suspensions, the cells were centrifuged, digested by aqua regia solution overnight, and diluted. ICP-OES was performed to determine the Fe content in the cell samples. To further confirm the cellular uptake of the Fe_3O_4/Au DSNFs, Prussian blue staining was employed. Similar to the ICP-OES analysis, Raw264.7 cells were treated with the Fe_3O_4/Au DSNFs for 6 h, then the cells were washed 3 times with PBS, and stained by a Prussian blue solution. Afterwards, the stained cells were observed by phase contrast microscopy.

Transwell Assay

In total, 5×10^5 4T1 cells were seeded in a 24-well plate, and a certain amount of Raw264.7 cells were seeded in a 6-well plate. DMEM was prepared with Fe_3O_4/Au DSNFs ([Au] = 3 mM) and added to a group of well plates of Raw264.7 cells and incubated for a total of 6 h. The other group was left untreated and used as a control. After incubation, the cells were washed with PBS and this was repeated multiple times to remove excess material. The experimental and control cells were treated with EDTA-free trypsin (sterile PBS) for 5–10 minutes, centrifuged to remove the supernatant, resuspended, and dispersed in a high-sugar medium without serum, and two groups were counted. Cells were seeded into the upper chamber, and each chamber was seeded with 200 μL of cell suspension. The plate was divided into four groups of controls, namely: complete medium in which macrophages did not have 4T1 cells, macrophages loaded with nanocomposite materials with no complete media in which 4T1 cells existed, macrophages with complete culture with 4T1 cells, and basic, nanocomposite-supported macrophages with a complete medium in which 4T1 cells were present. The inoculated Transwell plates were placed in a 37 °C incubator for 18 h.

After the incubation, the upper and lower chambers were washed several times with sterile PBS, and 500 μL of tissue fixing solution was added to each well to treat Raw264.7 cells for 15–30 min. A crystal violet solution with a mass fraction of 0.1% in methanol was prepared. After the fixation, we washed the upper and lower chambers with PBS multiple times, added 1 mL of crystal violet solution, stained for 30 min, and then washed with sterile PBS multiple times after the end. After treatment, we wiped the upper cells of the chamber gently with a moist cotton swab, taking care not to break the membrane with too much force. Finally, the bottom of the upper layer of the chamber was placed under a phase contrast microscope to randomly take 10–15 visual fields to count.

Detection of Raw264.7 Surface Antigen:

To identify the performance of Raw264.7 cells after uptake of the Fe_3O_4/Au DSNFs, surface antigens of the Raw264.7 cells were evaluated by flow cytometry (BD FACS Calibur, USA). Concisely, Raw264.7 cells were treated with the Fe_3O_4/Au DSNFs for 6 h, then the cells were washed 3 times with PBS, and stained with fluorescence conjugated antibodies CD80 and CD206 for 30 mins under the condition of 4 °C in a dark room. After

three washes with PBS, the stained Raw264.7 cells were ready for a flow cytometry analysis. Raw264.7 cells cultured in complete DEME medium for 6 h were used as the control group. Then, the changes of the surface antigen of the disposed Raw264.7 cells were analyzed by flow cytometry.

In Vivo MR/CT Imaging

Male 4~6 weeks old ICR mice (15-20 g, Shanghai Sleek Lab Animal Center, Shanghai, China) were used for mouse breast tumor MR imaging. A total of 2 × 10^6 4T1 cells per mouse were injected into the left leg to build the breast tumor model. When the tumor size of the mouse had reached 0.45~0.75 cm^3, Raw264.7 cells mediated Fe_3O_4/Au DSNFs ([Fe] =100 μg, in 0.2 mL PBS) were injected into each mouse through the tail vein. Another group of mice received the similar dose of free Fe_3O_4/Au DSNFs as controls. The in vivo 4T1 tumor imaging studies were measured by the MR system (Shanghai Niumag Corporation, Shanghai, China). The T1-weighted MR images of the mice were collected before and after injection of the tested nanomaterials at different time points.

For in vivo CT imaging, the MA@Fe_3O_4/Au DSNFs and Fe_3O_4/Au DSNFs ([Au] = 40 mM, in 200 μL PBS) were injected into each mouse via the tail vein. CT images of tumors were collected at different time points (0, 15, 30, 45, 60, 75, and 90 min, respectively) post intravenous injection by a GE LightSpeed VCT Imaging System with the parameters similar to those mentioned above. The tumor CT values were then quantified.

Statistical Analysis

A one-way ANOVA statistical analysis was used to analyze the significance of the experimental data. A p value of 0.05 was selected as the level of significance, and the data were indicated with (*) for $p < 0.05$, (**) for $p < 0.01$, and (***) for $p < 0.001$, respectively.

Results and Discussion

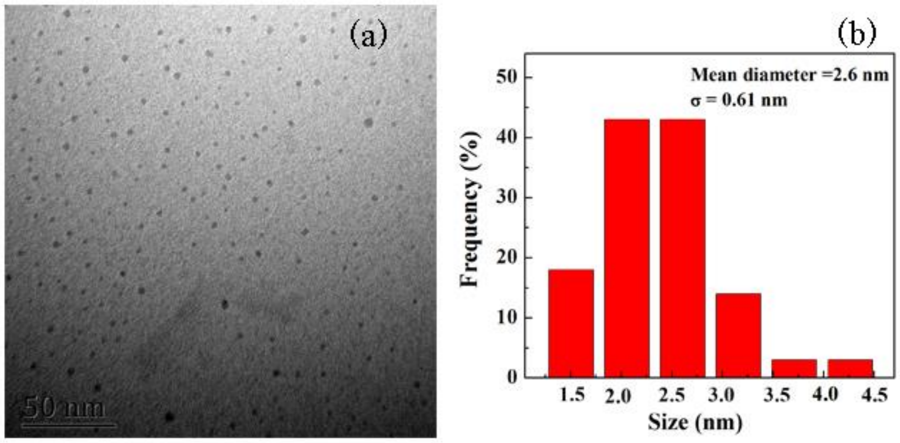

Figure S1. (**a**) TEM image and (**b**) the average size distribution histogram of USIO NPs.

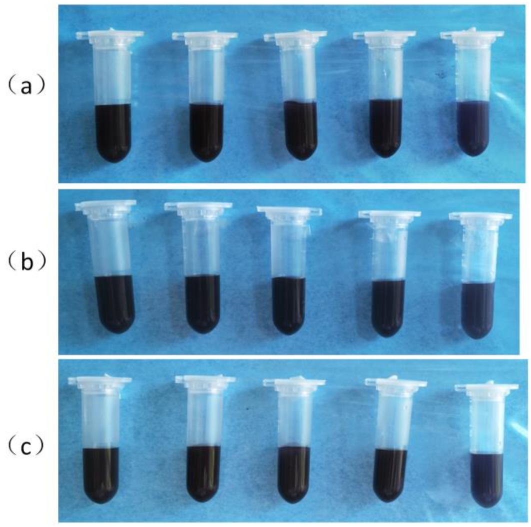

Figure S2. Digital photos of Fe_3O_4/Au DSNFs with different molar ratios of Fe/Au (from 1:1 to 6:1), (**a**) 3 d (**b**) 5 d (**c**) 7 d.

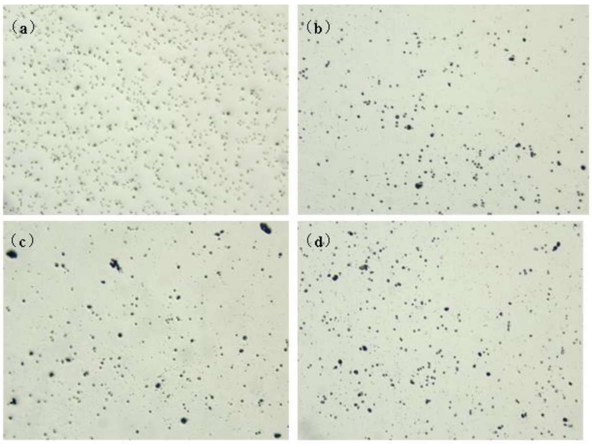

Figure S3. MA@Fe_3O_4/Au DSNFs with different Fe concentrations（0, 0.44, 0.88, and 1.76 mM）to incubate with Raw264.7 for 6 h and stain with Prussian blue reagent.

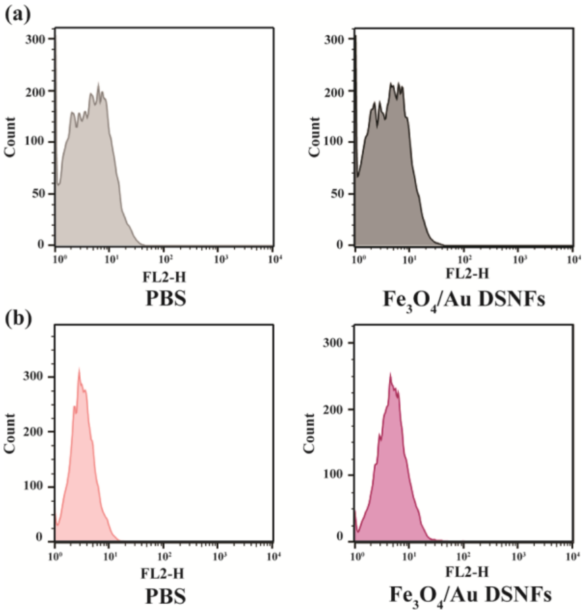

Figure S4. Detection of specific CD markers in Raw264.7 by flow cytometric analysis. Raw264.7 cells were stained with fluorescence conjugated antibodies fluorescein phycoerythrin conjugated rat anti mouse (**a**) CD206 and (**b**) CD80.

References

1. Hao, X.; Xu, B.; Chen, H.; Wang, X.; Zhang, J.; Guo, R.; Shi, X.; Cao, X. Stem cell-mediated delivery of nanogels loaded with ultrasmall iron oxide nanoparticles for enhanced tumor MR imaging. *Nanoscale* **2019**, *11*, 4904–4910, doi:10.1039/c8nr10490e.
2. Liu, H.; Xu, Y.; Wen, S.; Zhu, J.; Zheng, L.; Shen, M.; Zhao, J.; Zhang, G.; Shi, X. Facile Hydrothermal Synthesis of Low Generation Dendrimer-Stabilized Gold Nanoparticles for in Vivo Computed Tomography Imaging Applications. *Polym. Chem.* **2013**, *4*, 1788–1795.
3. Li, J.; Hu, Y.; Yang, J.; Wei, P.; Sun, W.; Shen, M.; Zhang, G.; Shi, X. Hyaluronic acid-modified Fe_3O_4@Au core/shell nanostars for multimodal imaging and photothermal therapy of tumors. *Biomaterials* **2015**, *38*, 10–21, doi:10.1016/j.biomaterials.2014.10.065.
4. Lu, S.; Li, X.; Zhang, J.; Peng, C.; Shen, M.; Shi, X. Dendrimer-Stabilized Gold Nanoflowers Embedded with Ultrasmall Iron Oxide Nanoparticles for Multimode Imaging-Guided Combination Therapy of Tumors. *Adv. Sci.* **2018**, *5*, 1801612, doi:10.1002/advs.201801612.
